# Ischemic Stroke in Young Female Adults Caused by Carotid Webs: A Case Series From Rashid Hospital, Dubai

**DOI:** 10.7759/cureus.77953

**Published:** 2025-01-25

**Authors:** Suhaila Ahmed, Mohammad Zaid, Ala E Nwilati, Mamoun Shafaamri, Mohammad S Faghih, Avdullah Qafani, Ayman Al-Sibaie, Michael G Shahoud, Masoud Shafiei, Deena M Al Qedrah

**Affiliations:** 1 Vascular Surgery, Dubai Health, Dubai, ARE; 2 Urology, Dubai Health, Dubai, ARE; 3 Radiology, Dubai Health, Dubai, ARE

**Keywords:** carotid endarterectomy, carotid webs, cryptogenic stroke, fibromuscular dysplasia, vascular anomalies

## Abstract

Carotid webs are rare vascular anomalies increasingly recognized as significant contributors to ischemic strokes in young, otherwise healthy adults, particularly females. These subtle vascular malformations are often underdiagnosed due to their inconspicuous appearance on standard imaging. This case series presents three young female patients, aged 30 to 39, who presented with acute ischemic strokes attributed to carotid webs. Through comprehensive stroke evaluation, including advanced imaging techniques and timely interventions, the presence of carotid webs was confirmed. Each case underwent surgical intervention with favorable outcomes. This series aims to raise awareness about the clinical significance of carotid webs in young women presenting with cryptogenic strokes.

## Introduction

Carotid webs are rare vascular anomalies that significantly elevate the risk of ischemic strokes, especially in young individuals lacking typical vascular risk factors [1]. These shelf-like fibroelastic projections, often found at the carotid bifurcation, disrupt normal blood flow and promote thrombosis, increasing the likelihood of embolic events. As a subtype of fibromuscular dysplasia (FMD), carotid webs can mimic common stroke etiologies, making their recognition essential for effective management, particularly in cases of cryptogenic stroke [2]. If left untreated, symptomatic strokes can recur in up to 20% of patients within two years [3].

Typically, carotid webs manifest as variants of the internal carotid artery bulb, where the intimal layer protrudes into the lumen, sometimes extending into the common carotid artery [4]. Digital subtraction angiography (DSA) is the gold standard for diagnosis, while color-flow duplex Doppler and transcranial Doppler provide valuable supplementary insights [5]. However, carotid webs may not be visible on computed tomography (CT) or magnetic resonance angiography (MRA), potentially leading to underdiagnosis. Post-mortem studies have indicated an incidence of 38% for carotid webs, compared to a prevalence of only 1.0% to 1.2% in patients who underwent CT angiograms for suspected ischemic changes [6].

Research on carotid webs, particularly their role in stroke pathogenesis among young women, remains limited. A significant gap exists in understanding sex differences in younger stroke patients, particularly regarding the influence of oral contraceptives (OCPs), which have been linked to a recurrent stroke rate of 4.5% [7].

Given their rarity and potential for misdiagnosis, maintaining a high index of suspicion for carotid webs is crucial, especially in young women presenting with ischemic strokes, cryptogenic strokes, or progressive stroke syndromes. This case series discusses three women who were admitted with acute ischemic strokes and progressive neurological deficits, emphasizing the importance of considering carotid webs in the differential diagnosis for young female patients, particularly those with unexplained neurological symptoms. By raising awareness of carotid webs as a significant source of atypical strokes, this study emphasizes the need for thorough stroke evaluations to ensure timely diagnosis and appropriate management.

## Case presentation

This retrospective case series includes three young female patients admitted to Rashid Hospital between August and September 2024, all presenting with acute ischemic strokes. Each patient underwent comprehensive stroke evaluation, including CT brain scans, CT angiography, carotid ultrasound, and subsequent treatment with carotid endarterectomy. Case one involved a 30-year-old female with sudden-onset left-sided weakness, revealing a right-sided ischemic stroke and risk factors for cerebral small vessel disease. Despite urgent endovascular intervention within six hours, she developed a large luminal infarct that did not improve. Case 2 presented a 39-year-old female with similar progressive deficits; however, timely endovascular treatment resulted in remarkable symptom recovery. In Case 3, a 35-year-old female with traditional atherosclerotic risk factors exhibited progressive stroke syndrome. Although treatment was initiated, she too had already sustained a significant infarct, limiting the outcome to halting further progression.

Case one

A 30-year-old right-handed Nigerian female presented to the emergency department on September 8, 2024, with sudden onset of slurred speech, right-sided weakness, and a generalized headache (severity four out of ten) that had occurred within the previous hour. Her symptoms began after she went to the restroom, and her friend confirmed she was in a normal state beforehand. She denied any seizures, loss of consciousness, or history of similar episodes. Her medical history included an ocular injury five years ago, which caused temporary visual disturbances, but she had no significant surgical history or family medical history. She was on weight reduction medication, a non-smoker, and employed as a chef.

On examination, she was vitally stable. Her body mass index (BMI) was 32 kg/m² (obese). She was alert with a Glasgow Coma Scale (GCS) of 15 but had global aphasia, dysarthria, anisocoria (right pupil 3 mm, left pupil 2 mm), right homonymous hemianopia, and right upper motor neuron facial weakness. Neurological examination revealed 0/5 motor strength in both right limbs, with equivocal plantar reflexes bilaterally and reduced sensation on the right side. The cerebellar exam was normal. 

**Figure 1:**
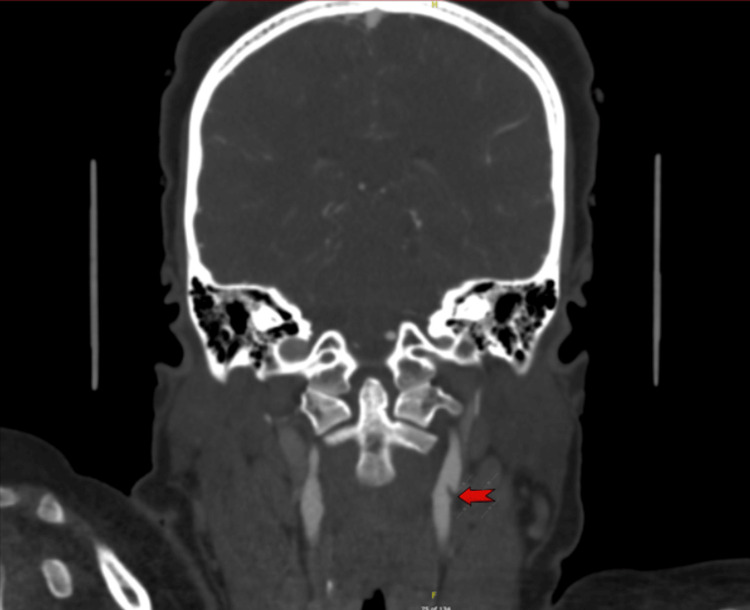
CT angiography (Coronal view) Red arrow showing irregular hypodensity at the left internal carotid artery origin, suggestive of a web or plaque.

**Figure 2:**
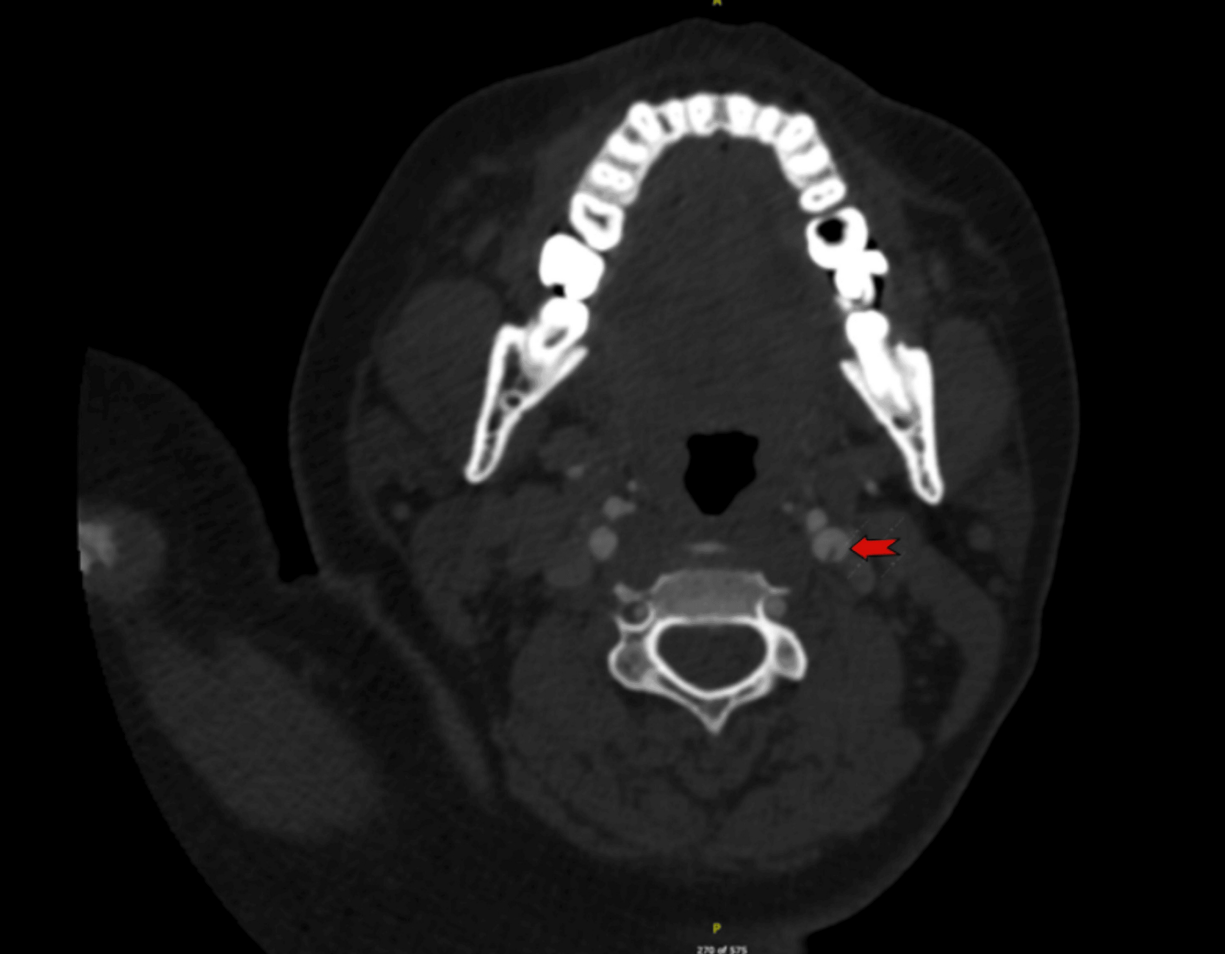
CT Angiography (Axial view) Red arrow showing irregular hypodensity at the left internal carotid artery origin, suggestive of a web or plaque.

Lab results showed low hemoglobin (10.6 g/dL), elevated C-reactive protein (CRP) of 30.1 mg/dL, and an increased erythrocyte sedimentation rate (ESR) of 67 mm/hr. Thyroid function tests were normal, and her lipid profile revealed slightly low high-density cholesterol (HDL) of 48 mg/dL but normal cholesterol levels. Coagulation studies showed a prolonged prothrombin time of 14.9 seconds and an elevated international normalized ratio (INR) of 1.25, with normal activated partial thromboplastin time (APTT). A CT brain revealed an acute infarct in the left middle cerebral artery (MCA) territory. CT angiography was done, as seen in Figure 1 and Figure 2.

ECG showed sinus rhythm with normal parameters, and echocardiography was also normal. The patient received tissue plasminogen activator (tPA), but oral bleeding from her left molar necessitated intubation. Mechanical thrombectomy and thrombolysis were performed without complications. A follow-up CT brain scan performed on October 8, 2024, showed no hemorrhagic transformation; the patient continued on antiplatelet therapy. The carotid ultrasound showed no significant findings A later CT brain stroke and angiography identified a distal common carotid web extending into the left internal carotid artery. 

**Figure 3:**
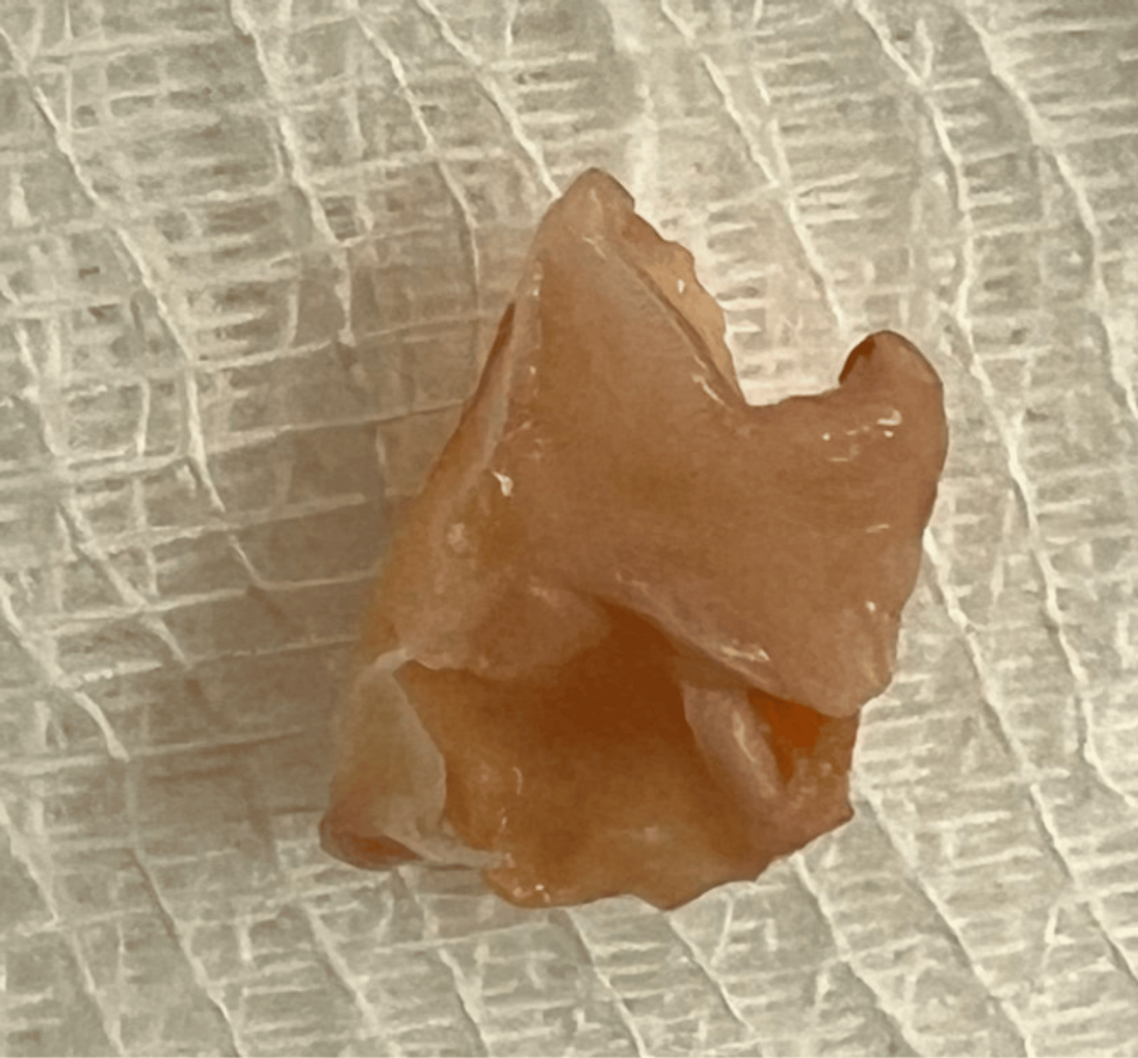
Resected carotid web The specimen shows the resected carotid web

On August 19, 2024, she underwent resection of the carotid web with synthetic patch plasty. The specimen obtained is seen in Figure 3. 

Postoperatively, she was stable with no neurological deficits. A bedside ultrasound done before discharging her showed a patent right internal carotid artery and no thrombosis. The patient was discharged with instructions to continue aspirin therapy and to return for a follow-up in three months for a carotid ultrasound assessment.

Case two

A 39-year-old Filipino female with no known comorbidities presented to the emergency department on August 23, 2024, after experiencing dizziness and left-sided weakness upon waking at 6:00 AM. She had been last seen in a normal state at 11:00 PM the previous night. The patient denied any loss of consciousness, headaches, or neck pain, although she reported right-sided neck pain for two to three days before admission. Her past medical history included anemia, for which she had taken iron and folic acid supplements. 

On examination, she was vitally stable, with a BMI of 24.2 kg/m² (normal weight). Neurologically, she was alert and responsive, but responses were delayed. She had gaze deviation to the right, slurred speech, left-sided homonymous hemianopia, left facial asymmetry, and motor weakness (3/5 in the left upper limb, 4/5 in the left lower limb). 

**Figure 4:**
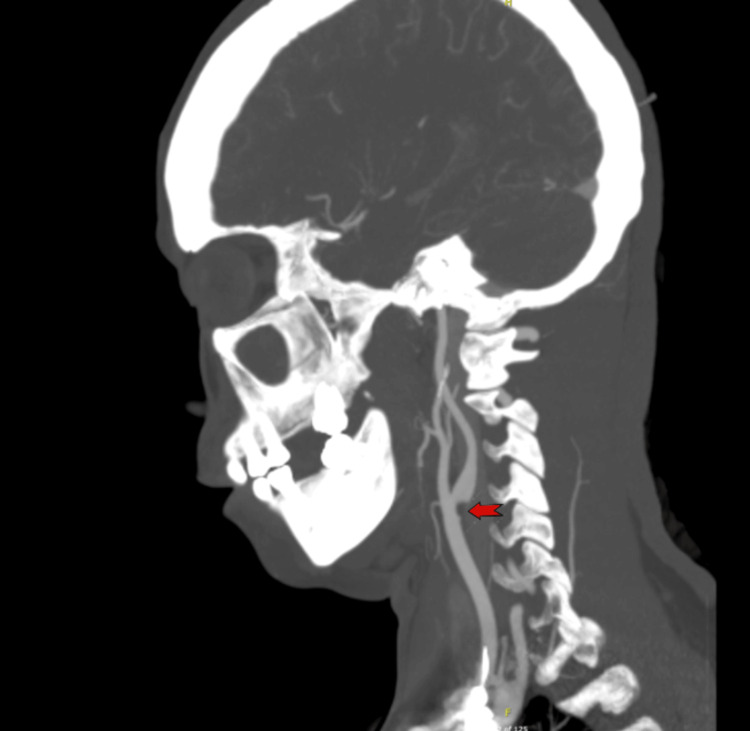
CT angiography (Sagittal view) It revealed hypodensities in the right frontal and temporal regions and occlusion of the superior and inferior branches of the M3 segment of the right middle cerebral artery, and a focal filling defect at the carotid bulb/origin of the right internal carotid artery as shown by the red arrow, likely due to thrombus formation from dissection.

The initial diagnostic investigations included a CT brain scan and a CT cerebral angiogram, shown in Figure 4.

ECG showed sinus rhythm with normal parameters, and echocardiography was also normal.

**Figure 5:**
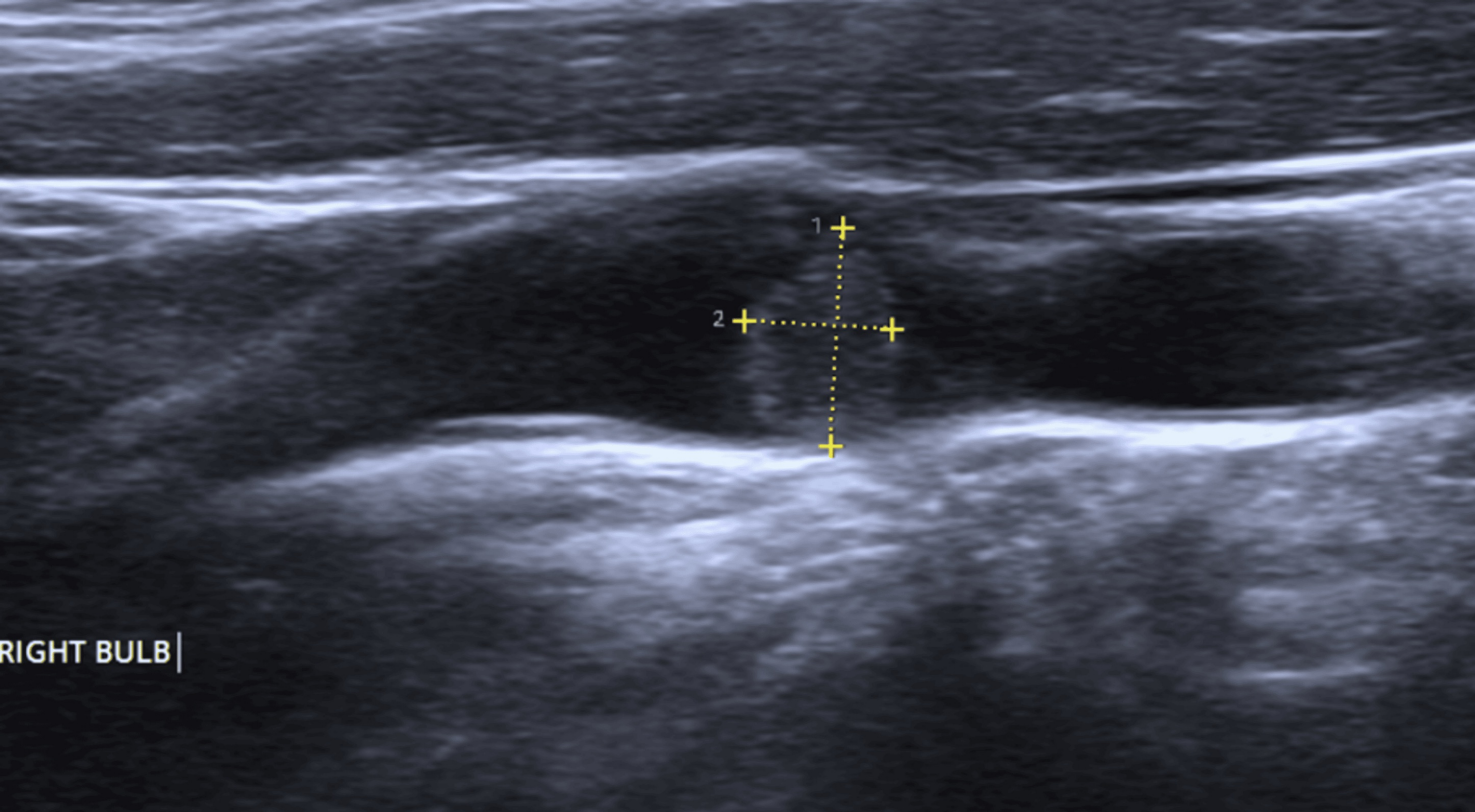
Carotid artery ultrasound Showing a soft plaque measuring 6.4 mm x 4.3 mm in the right carotid bulb.

Laboratory results revealed low pH (7.334), elevated partial pressure of carbon dioxide (pCO2) of 45.4 mmHg, low partial pressure of oxygen (pO2) of 24.1 mmHg, elevated hemoglobin (15.1 g/dL), and high glucose levels (145 mg/dL). The lipid profile was within acceptable ranges, with total cholesterol at 181 mg/dL, triglycerides at 71 mg/dL, HDL at 68 mg/dL, and low-density lipoprotein (LDL) at 99 mg/dL. The coagulation profile showed normal prothrombin time (13.4 seconds), INR (1.06), and APTT (29.4 seconds), but revealed a protein S deficiency at 31%, increasing thromboembolic risk. A follow-up neuroimaging confirmed a right frontal infarction. A carotid artery doppler was done as seen in Figure 5. 

Due to the risk of recurrent stroke, a right carotid endarterectomy with synthetic patch plasty was scheduled for September 2, 2024. The operation was uneventful and postoperatively, the patient remained neurologically intact, exhibiting no signs of neck hematoma or any other complications. A bedside ultrasound done before discharging her showed a patent right internal carotid artery and no thrombosis. She was subsequently discharged in stable condition following her postoperative recovery.

Case three

A 35-year-old African female presented to the emergency department on August 30, 2024, with sudden-onset left-sided body weakness that began at 8:00 AM. The weakness affected her face, upper limb, and lower limb. She had been asymptomatic the previous night. She denied any history of fever, diabetes, hypertension, smoking, alcohol use, illicit drugs, or oral contraceptive pill use. There was no family history of stroke or cardiovascular disease, and she was a single mother with no known allergies.

On examination, she was vitally stable. Her BMI was 31.2 kg/m² (obese). She was fully alert and verbalizing but presented with left-sided facial asymmetry due to an upper motor neuron lesion. Motor examination revealed dense left hemiplegia, with 1/5 power in the left upper limb and 2/5 in the left lower limb. She exhibited left hemineglect and a left visual field cut, while her right-sided strength was normal (5/5), and there was no sensory loss. 

**Figure 6:**
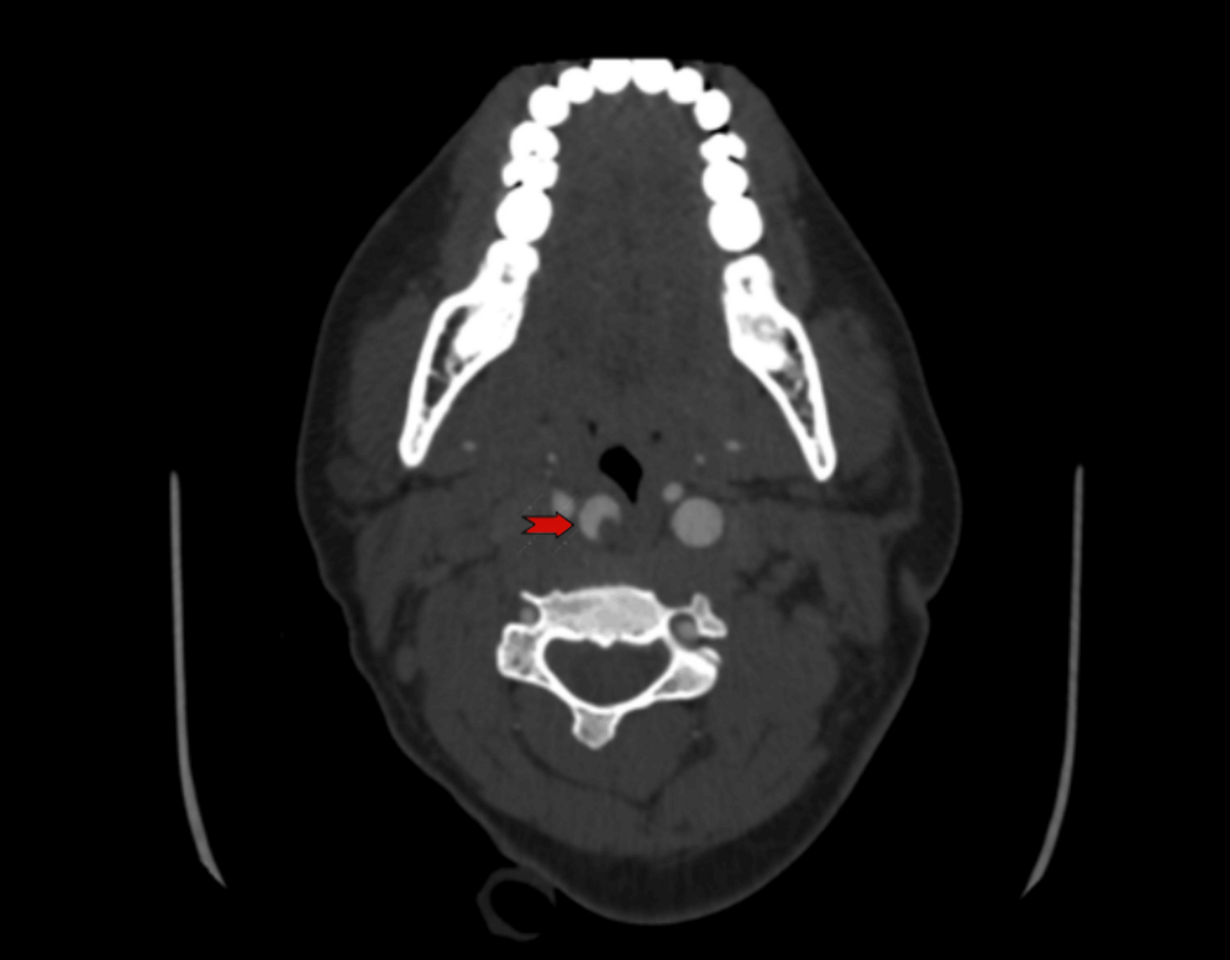
CT angiography (Axial view) Revealed acute occlusion at the right M1-M2 junction, with a soft thrombus at the origin of the right internal carotid artery causing 45% occlusion (red arrow).

A CT brain scan indicated an evolving ischemic stroke in the right basal ganglia, caudate nucleus, and right temporoparietal and frontal lobes. CT angiography was done, as shown in Figure 6. 

She underwent a mechanical thrombectomy and the thrombus was successfully removed from the right M1-M2 junction, restoring blood flow. Post-procedure imaging confirmed reperfusion of the affected territories. The patient was closely monitored in the intensive care unit for 48 hours and was started on dual antiplatelet therapy with aspirin and clopidogrel, along with statin therapy for secondary stroke prevention.

**Figure 7:**
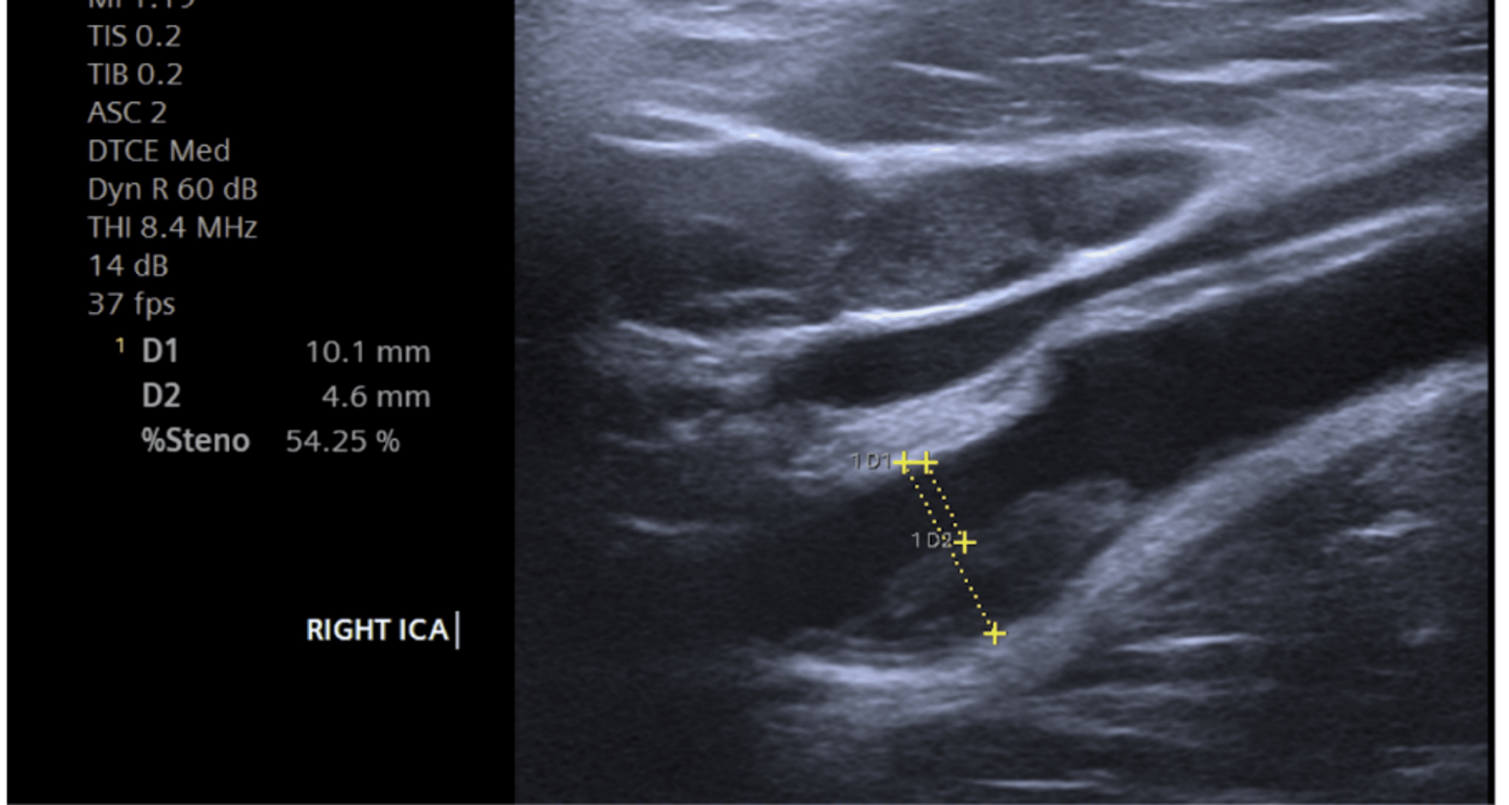
Carotid artery doppler ultrasound Revealed a homogeneous soft plaque at the proximal right internal carotid artery with 50% stenosis.

On August 31, 2024, follow-up CT showed hemorrhagic transformation of the infarct with mild mass effect and a 4 mm midline shift. She was started on low molecular weight heparin for deep vein thrombosis prophylaxis and continued on atorvastatin and physiotherapy. A carotid artery Doppler ultrasound was done as seen in Figure 7. 

A transthoracic echocardiogram on the same day was normal and the Holter monitor recorded sinus rhythm with no significant arrhythmias or heart blocks. Her blood investigations revealed prediabetes (HbA1c 5.8%), anemia with low iron levels (iron 26 ug/dL), and elevated CRP of 5.7 mg/dL. Her lipid profile was normal and there were no significant findings on thrombophilia screening, although her protein S level was low at 49%. 

On September 9, 2024, she underwent a right carotid endarterectomy with synthetic patch plasty due to recurrent thrombus formation. No new neurological deficits were noted postoperatively.

A bedside ultrasound done before discharging her showed a patent right internal carotid artery and no thrombosis. The patient was discharged on September 11, 2024, and was scheduled for a follow-up in one month for a carotid ultrasound and dressing changes every three days until suture removal.

The table below (Table 1) presents a comprehensive comparison of the three clinical cases diagnosed with carotid web, detailing patient demographics, presenting symptoms, imaging findings, treatments, and postoperative outcomes.

**Table 1:** Table comparing the three cases BMI: body mass index, MCA: middle cerebral artery, tPA: tissue plasminogen activator

Feature	Case 1	Case 2	Case 3
Demographics	30-year-old Nigerian female, obese (BMI 32 kg/m²)	39-year-old Filipino female, normal weight (BMI 24.2 kg/m²)	35-year-old African female, obese (BMI 31.2 kg/m²)
Presenting Symptoms	Sudden onset slurred speech, global aphasia, right-sided weakness, generalized headache, right homonymous hemianopia	Sudden onset slurred speech, left-sided weakness, left homonymous hemianopia, left facial asymmetry, gaze deviation to the right	Sudden onset left-sided body weakness, left hemiplegia, left visual field cut, left-sided facial asymmetry, left hemineglect
Imaging findings	CT Brain: Acute infarct in left MCA territory, CT angiography: Irregular hypodensity at left internal carotid artery origin (carotid web or plaque)	CT Brain: Acute right frontal infarction, CT Angiography: Occlusion of M3 segment branches of right MCA, filling defect at right internal carotid artery (thrombus)	CT Brain: Evolving ischemic stroke in right basal ganglia, caudate nucleus, and temporoparietal and frontal lobes, CT Angiography: Acute occlusion at right M1-M2 junction
Treatment	tPA administration, mechanical thrombectomy, carotid endarterectomy with synthetic patch plasty	Carotid endarterectomy with synthetic patch plasty	Mechanical thrombectomy, dual antiplatelet therapy (aspirin and clopidogrel), carotid endarterectomy with synthetic patch plasty
Postoperative Outcome	No complications post-surgery; stable post-procedure with antiplatelet therapy	No complications post-surgery, discharged in stable condition	No complications post-surgery; discharged in stable condition

## Discussion

Carotid webs are increasingly recognized as a significant but underdiagnosed cause of ischemic strokes, particularly in young women [8]. The three cases presented in this series illustrate the diverse presentations and clinical challenges associated with diagnosing carotid webs in patients who do not exhibit typical cerebrovascular risk factors. The subtleness of carotid webs on standard imaging techniques often leads to missed diagnoses, emphasizing the importance of a high index of suspicion in young patients presenting with cryptogenic strokes [9].

The management of carotid webs involves timely diagnosis and intervention to mitigate the risk of recurrent strokes. The gold standard for diagnosis remains DSA, which effectively visualizes the structural abnormalities associated with carotid webs [10]. In addition, complementary imaging techniques, such as color-flow duplex Doppler ultrasound and transcranial Doppler, can enhance detection and provide insights into blood flow abnormalities [11]. Surgical intervention, particularly carotid endarterectomy, has proven effective in excising the web and preventing subsequent ischemic events. In our cases, the prompt surgical management not only resulted in successful excisions of the webs but also avoided postoperative complications, highlighting the procedure's efficacy [12].

Despite advancements in imaging and surgical techniques, gaps in understanding the role of carotid webs in stroke pathogenesis persist. Further research is needed to investigate the associations between carotid webs and other potential risk factors, including the impact of oral contraceptives in young women [13]. Prospective, multi-centered studies focusing on these atypical vascular anomalies will be essential for developing standardized diagnostic criteria and treatment guidelines.

## Conclusions

Carotid webs are a significant yet underdiagnosed cause of ischemic strokes, particularly in young women, as highlighted by the presented cases. These cases emphasize the importance of prompt recognition and timely intervention to reduce the substantial risk of recurrent strokes, which, if left untreated, can reach up to 20% within two years. Despite advancements in imaging, identifying carotid webs remains challenging, as standard modalities like ultrasound and CT scans may not always provide definitive diagnoses. This emphasizes the need for advanced imaging techniques and clinical vigilance to ensure accurate detection. By addressing these diagnostic challenges and prioritizing early surgical management, clinicians can improve outcomes and reduce stroke recurrence in this vulnerable population.
